# Dental Education

**Published:** 1890-06

**Authors:** C. Stoddard Smith


### DENTAL EDUCATION.[1]

BY C. STODDARD SMITH, D.D.S.

TIME was when the seeker after dental knowledge, upon the payment in lawful money of one hundred or two hundred dollars to a so-called preceptor, could be assured of a complete initiation into the mechanisms, mysteries, and secrets of the dental art in the space of a few short weeks, or months at furthest. Peeping through the crack of the laboratory door, he watched his preceptor as he mixed and inserted the "royal mineral succedaneum" (as the quacks of that day denominated amalgam), or "plugged" the carious molar with "gold leaf," introduced by smooth-bore instruments, or

---

[1] Read before the Chicago Dental Club, March 24, 1890.

fixed the key on the dental organ which had ached. Secure in the private recesses of the laboratory, with doors closed so that the prying and non-paying world should not share in his good fortune, he became acquainted with the mysteries of wax impressions, casts, dies, and soldering lamps. It was generally believed that the greatest effort and skill was required to master the secret processes of what is now somewhat pedantically denominated prothetic or prosthetic dentistry, while the art of saving the natural teeth was very easily acquired. To be sure, the student had to absorb and assimilate the fact that arsenic would "pizen" nerves; but contour fillings were an unknown quantity, and "gum-biles" were a pathological condition beyond the reach of the therapeutical knowledge of those by-gone days. A few plain excavators and pluggers, a key, a pair or two of clumsy forceps, a book of gold, and a pound or so of amalgam, with a scant outfit for making metal plates, and possibly a rolling-mill, comprised the stock in trade of the most progressive dentist. Yet I have seen gold plates made by a dentist of that period that for fit and workmanship would equal any, and far surpass nineteen-twentieths of the work of later years.

But that day has long since passed. Dentistry has outgrown and cast off its swaddling-clothes, passed through the period of adolescence, and emerged into the arena of a recognized profession.

Dental education in its modern phases is the subject we have before us for consideration this evening. What should it be, and how should it best be accomplished?

The dental student of to-day is easily resolvable, in origin, to one of two classes. The country youth, to whom a dollar resembles a cart-wheel in size, having discovered that he has just a little hole in one of his teeth, visits the village dentist, and is informed that there are a dozen cavities; and the amount of the bill, and the apparent ease with which the money is secured by the dentist, lead the patient to believe that a dental office is a "heap better than a farm," or "tending store," and is, in fact, a veritable bonanza, a soft snap, a philosopher's stone, or a Midas touch, which shall literally turn base metal to gold, and gold into greenbacks with about one thousand per cent. profit. He casts longing eyes on the environment of the practitioner, wonders how long it would take him to "learn the trade," and soon determines to reach the wished-for goal, or die in the attempt. Another class is composed of the sons of well-to-do and ambitious parents, who, like the country youth, have been led to believe that the main work of the dentist is to take care of the money as it comes in; that the business is genteel as well as lucrative; and that it requires little time to learn it or capital to set up in it. The aspiring father sees in dentistry the short line to success, position, emolument, and most likely affluence for his offspring.

From both of these classes, as well as from other sources, come the rank and file of dental students to-day. But from whatever source they come, whatever motives actuate them, they one and all utter and re-utter the self-same query, "What is the least possible time and the smallest amount of money which it is necessary for me to devote before I can emerge a legitimate practitioner, and share in these golden rewards?"

Dental laws in nearly all the States have made it necessary that some sort of education shall be obtained; at least, that a diploma must be secured. These laws might with propriety have been entitled laws to promote the organization and operation of dental colleges. Certainly their effect has been to vastly multiply the number at least of these institutions. Armed with a diploma from one of them, the student possesses the "open sesame" to the mine of wealth he thinks he is about to put his pick into. He can defy examining boards, and by putting out his shingle with a D.D.S. attachment easily secure the prize.

Though the mallet has done much in private practice to bring the student from behind the laboratory door, and enable him to view the actual operation of filling, yet the patient objects to his presence as a rule, and the various pathological conditions, the proper formation of cavities, and many other matters are nearly as far from his reach and observation as ever. Few patients will allow him to operate, even in the simplest cases. Further, few preceptors have the ability, the gift, or the time to teach the student, even had they the desire; and in many cases their main idea is to obtain as much in the way of service as is possible, with as little of effort on their part as may be. The dental college, properly officered, equipped, and conducted, changes all this. From the moment the student enters its doors he is in possession of facilities and advantages which years of private pupilage could never afford him. He has systematic and thorough instruction by didactic lectures; he has skilled instructors ever ready to encourage and aid his efforts, and to show him what to do and how to do it; he has patients awaiting his manipulations, who come with the expectation of such comparatively unskilled service. If studious, industrious, and energetic in using his opportunities, he can learn more in six months than he possibly could in two years under any preceptor, no matter how willing or competent to teach.

The dental college, then, must be regarded as the chief educational agency of the dental profession. It must, of course, be supplemented by text-books, and if then is added the advantage of a private preceptorship, so much the better. When a man starts out in business of any kind, he naturally seeks to secure that which he must have as his stock in trade where he can obtain it to the best advantage. The dental student, whose dental knowledge is to be his stock in trade, is no exception, and, recognizing the soundness of the above conclusions, he seeks the dental college naturally and unavoidably. If we trace his career through these institutions, see what he actually does, or tries to do, what he accomplishes, and how he is equipped for his chosen profession when he emerges, we may, by comparing the actual process with an ideal one, be able to arrive at a conclusion as to whether dental education, as now conducted, is what it ought to be or what it might be.

All dental colleges of repute profess to require a preliminary examination in English branches. In other words, it is assumed that a man who is to occupy a professional position should be possessed of a common-school education at least, and have an intelligent knowledge of common branches. It ought, at least, to be understood that he will be required to show a decent knowledge of what the backwoods school-director termed "the three R's," to wit, "readin' 'ritin', and 'rithmetic," and it might not be too much to require a rudimentary knowledge of "spellin'" as well. But in how many colleges may we suppose this requirement to be anything more than a hollow mockery? Since this paper was begun the writer had it direct from a matriculate of a college which claims to stand high, that no examination whatever was required of him. It is probably the case, in at least a great many instances, that the process of matriculation consists of the transferring of a "V" from the pocket of the candidate to that of the dean, and the inscribing of the name of the applicant upon the roll of matriculates, unaccompanied by any such formalities as finding out whether the student knows anything whatever.

We will suppose that the student is "green," and enters at the fall term. He is at once plunged into an atmosphere entirely unknown to him. He hears terms of which he knows not the meaning, allusions and descriptions which he does not understand; he gets a dose of one or two subjects in the morning and of one or two more in the evening, with an intermediate admixture of practical operations of some sort. What wonder, then, if at the end of three months he becomes confused and bewildered? Education, in its literal and proper sense, means a drawing out,—an attempt to evolve or bring out the capacities of the individual. Dental education, as now conducted, seems to be largely a pouring in or a filling up process. It begins in this manner and it continues on this line to the end, and too often, we fear, the attempt is made to pour in too fast, if not too much. The two terms of five or six months each is far too short a time to teach thoroughly all that is attempted in the curriculum of the principal dental colleges of to-day. It is not possible that the average student should in that brief time obtain more than a smattering of the many branches. It is well, therefore, and we regard it as a great step in advance, that this time is to be lengthened to three years. And when we say three years, we mean thirty-six months of solar time, and not three years of five or six months each. That surely is none too long to gain even a superficial knowledge of all these subjects. All honor to those men who have worked to effect this end, whose efforts have at last set the date when this reform is to go into effect; and now let every individual member of the profession endorse and sustain it by refusing to receive any student who will not pledge himself to spend this length of time; and let every State Board enforce it—as they have the power to do—by refusing to recognize any college as reputable which does not require it, or to admit to an examination any candidate who cannot show satisfactory proofs that he has studied for three years. Both of these things the Illinois State Board has done, but, so far as we know, no other Board has done so.

This is, however, a digression; we were discussing dental education as it is. The attempt to teach all branches during a six-months' term is a baneful effort to cram the student. The graded course, in which the studies of the first and second year are separated, is a great advantage, and should be generally adopted, as it is now by many or perhaps most of the better colleges. But, notwithstanding this, there is, we fear, a constant attempt to do too much. It is quite impossible to make a pint measure hold a quart. It is equally impossible to make complete anatomists and chemists and therapeutists and metallurgists and physiologists and microscopists out of a class of average students,—that is, to make *all* of them thorough in *all* these branches. Yet each professor goes to work as if his object was to make a specialist in his particular line. He seems to forget that he is but the part of a whole. Whether dentistry is a specialty of medicine or not, is a question foreign to this paper. The attempt to make it such by first demanding an M.D. degree has not been successful, and is not likely to be. Nor is the attempt to carry the instruction in purely medical and theoretical branches to an extreme, as it is my opinion is too often done, likely to be any more of a success. The student enters upon his second year face to face with the fact that he must master the remaining branches, or fail to secure his coveted prize. He *must* fit himself for the dreaded examinations. To this all his efforts are bent. He plunges into the vortex overwhelmed by misgivings, and it is then that the cramming process begins in earnest. As the time for the dread ordeal approaches, he is treated like the Christmas turkey, whose neck is stretched to stuff his crop with grain by the forcing process, rammed down with a ramrod, irrespective of the fact that the pabulum of the previous day or week lies undigested and unassimilated. He finds that he cannot possibly do all that is required of him. He *must* neglect something. He manages to get in the required fillings, and his practical artificial case, in some way, but that examination ever haunts him as a spectre. Unless he shall succeed in passing it, he cannot go forth with his degree. The result is that he emerges accredited to the world as skilled and competent, when really his practical knowledge is that of the merest tyro. He bears the insignia of competency, but really he is entirely unprepared to grapple with the actual demands of practice. He begins at once to learn at the expense of the unfortunate public who may fall into his hands what he should have been taught while in college. He has learned imperfectly, and in many cases very indifferently, how to master the ordinary office practice; how to make a good gold filling, the *sine qua non* of successful practice; and as to the so-called prosthetic dentistry, he is in very many cases an utter failure, especially as to the despised but much-called-for rubber plates. In extracting teeth he is probably a butcher. In the very essential matter of dealing with the public, of conducting a practice, he has most likely never had any instruction whatever. He has to pay well in time and money for teaching which he supposed was to make him a dentist, but the tendency of which has all the time been to make a specialist of him. In this attempt there is no probability of any considerable success. If a man is to be a chemist or a microscopist, he will find that what he learns in the dental college is merely rudimentary after all.

This tendency of the teaching of the day is not peculiar to dental education. It is found in our common schools and literary colleges as well. The pupil in our common schools is taught a little English, a little German, a little music, a little clay-modelling, a little of this, and a little of that, with the result that of any of these branches only the merest smattering is obtained; not enough to be of the least practical use in the business of life, and meantime the time which should have been spent in developing the powers and making progress in the common English branches is frittered away, and the pupil ends his school-days with no specialty at all mastered, and with a painfully imperfect knowledge of reading, spelling, grammar, arithmetic, and the common and practical things he will need to know and to use in order to fill his place in life. If he aspires to a higher education, he perhaps spends his time on Greek and Latin and Calculus, the result being that, although bearing the degree of A.M., he is forced to earn his living by some ordinary and perhaps subordinate occupation.

> "For many a man comes home from school,
> A Greek and Latin, Hebrew fool."

Let us not be misunderstood. We do not decry or belittle education. We respect and admire it. We wish to have every man who is attempting to get it get all that he possibly can, on all subjects. Let him imbibe from the fountain of knowledge to his full capacity. But what we *are* contending for is that an education looking towards an object should be practical in its relation to that object. It is supposed that the object of dental education is to make *dentists;* not specialists of some sort or other. Therefore all his teaching should be mainly directed towards making a *dentist* of him. Let him learn as much more as his capacities, time, and opportunities will admit; but unless he succeeds in making a dentist of himself, his dental education must be a failure, no matter how much he may know of special branches. Much as we may magnify the profession of dentistry, the fact remains that nine-tenths of its practice is almost wholly mechanical. Anatomy and pathology and therapeutics, of course, are necessary branches; but dentists, as such, do not need to spend time on more than their main principles and the details which are connected with dental practice; when we come to simmer it down, the pathological conditions with which the dentist ordinarily has to deal are comparatively simple, and their treatment is equally so. The polychrests which he really needs in his materia medica are quite few in number, and all the multiplication of new remedies seems to amount practically to very little. If the specialists in dental therapeutics and materia medica will bring forth a certain and unobjectionable remedy for sensitive dentine, and inaugurate an era of really painless dentistry, they will accomplish more for the dentist and the public than in fifty years of research to secure some new and original and probably expensive substance with which to do what has for decades been as well and successfully done with old and tried remedies as it can well be.

Then, again, microscopy is a very interesting and instructive science. It should be taught in every dental college. But can a dental student profitably spend days or weeks of the precious time of his course in making sections of liver and lung and vegetables? A dozen illustrated lectures (such as Dr. Sudduth can give) will teach him more that he really needs to know, and in a more effective manner, very likely, than he could be taught by making sections without number.

Our main plea is, therefore, that dental education should consist more of practical teaching and practical work. More of this, and *no less* of theory than heretofore, we would gladly say. But life is short, and so is the time allotted to preparing for the great work of life. The struggle for existence is ever present. Capacity, time, and means, all are often limited, and if the result is that one or the other must be sacrificed, let us cut off the theoretical and stick to the practical.

As it appears to us, one great defect of even the most pretentious of dental colleges is a lack of a proper number of chairs, and also of demonstrators. In one college which professes to have considerably over two hundred students there are fifty chairs, and hardly six demonstrators. If the two hundred students are studying dentistry, it is to be supposed that they should have the use of chairs. The proportion of chairs to students named would give each student a chair once in three to four days, or an opportunity to perform thirty to thirty-five operations in a term. If some of them were juniors and not allowed to operate, the number might be increased to fifty or possibly sixty. But we submit that this is far too small a portion of the time to be devoted to practical operations. And as to demonstrators, it is perfectly plain to any one that knows anything about it that eight chairs are more than the average demonstrator can attend to properly. Yet it is believed that the facilities named are as good as any, and very likely exceptionally good. This is all wrong. If the college desires to do its duty by the student, it will provide such clinical facilities as will enable him to properly acquire the skill which he must have to sustain himself in practice.

But time would fail were we to enumerate the defects of the actual and the requirements of the ideal dental education. The subject is only outlined here.

In conclusion, let us utter one more word of caution, lest the purport of this paper be misunderstood. It is the attainment of but few to become eminent in more than one department of life. To develop all that there is in a man, and direct it in one line of action, is all that can ordinarily be hoped for. Let those who control the educational institutions of the profession ever remember that their object should be to make *dentists*; to send out men competent in diagnosis, thorough and skilful in manipulation, kind and sympathetic in their bearing towards their patients, able to command the respect and confidence of the community. Let this be their object, and not the inculcation of some special fad; and let the student be made to feel that this is the case, and not that he is liable to be despoiled of his time and money and then either plucked and cast adrift because he fails to grasp some technicality, or else turned loose with a meagre equipment for the struggle which in most cases will confront him. He will then, if he has the right stuff in him, feel encouraged to put forth his best efforts, and be far more likely to succeed. Let the standard be never so high, but let it not be the case that the real and the practical is lost sight of in a fruitless effort to grasp the ideal.

